# *Eventogram*: A Visual Representation of Main Events in Biomedical Signals

**DOI:** 10.3390/bioengineering3040022

**Published:** 2016-09-22

**Authors:** Mohamed Elgendi

**Affiliations:** 1 Department of Obstetrics & Gynecology, University of British Columbia, Vancouver, BC V6Z 2K5, Canada; moe.elgendi@gmail.com; Tel.: +1-604-600-4139; 2 Department of Electrical and Computer Engineering, University of British Columbia, Vancouver, BC V6T 1Z4, Canada

**Keywords:** quasi-periodic signals, time-series visualization, signal transformation, event detection, signal segmentation, time-domain representation, spatio-temporal analysis, pattern discovery, waveform recognition

## Abstract

Biomedical signals carry valuable physiological information and many researchers have difficulty interpreting and analyzing long-term, one-dimensional, quasi-periodic biomedical signals. Traditionally, biomedical signals are analyzed and visualized using periodogram, spectrogram, and wavelet methods. However, these methods do not offer an informative visualization of main events within the processed signal. This paper attempts to provide an event-related framework to overcome the drawbacks of the traditional visualization methods and describe the main events within the biomedical signal in terms of duration and morphology. Electrocardiogram and photoplethysmogram signals are used in the analysis to demonstrate the differences between the traditional visualization methods, and their performance is compared against the proposed method, referred to as the “*eventogram*” in this paper. The proposed method is based on two event-related moving averages that visualizes the main time-domain events in the processed biomedical signals. The traditional visualization methods were unable to find dominant events in processed signals while the *eventogram* was able to visualize dominant events in signals in terms of duration and morphology. Moreover, *eventogram*-based detection algorithms succeeded with detecting main events in different biomedical signals with a sensitivity and positive predictivity >95%. The output of the *eventogram* captured unique patterns and signatures of physiological events, which could be used to visualize and identify abnormal waveforms in any quasi-periodic signal.

## 1. Introduction

Periodogram, spectrogram, and wavelet methods are the current standard methods for visualizing and analyzing one-dimensional, quasi-periodic biosignals. Such visualizations generate temporal-spatial information in order to gain scientific insights into the physical measurement. These powerful visualization techniques play a key role in understanding large and complex signals. The task of a visualization algorithm is to translate the raw signals into a meaningful graphical representation to facilitate interpretation by the scientist. In many cases, sophisticated analysis techniques can help to extract essential information from the biomedical signals.

The periodogram is a frequency visualization of the processed signal, while the spectrogram and wavelet are time-frequency visualizations. However, these techniques do not provide detailed information of the dominant events in terms of morphology and duration. Many researchers attempted to visualize signals in the time domain [1,2,3,4,5,6,7,8]; however, this massive research effort did not lead to a generic, intuitive implementation framework that is specifically applied across different disciplines, including current clinical problems [9].

The aim of this paper is to introduce a new, simple, easy-to-use visualization algorithm that extracts the main events in signals, especially in one-dimensional, quasi-periodic biosignals such as electrogradiograms (ECGs) and photoplethysmograms (PPGs). These biosignals contain valuable information represented by physiological events. Once an event has been detected, the corresponding signal can be extracted and analyzed in terms of its amplitude (peak), morphology, energy and entropy distribution, frequency content, and intervals between events. Determining the dominant events within these biomedical signals that will provide more insight into the information of the signal can ultimately improve the development of more accurate diagnostic tools. The commonly used visualization methods known as the periodogram, spectrogram, and wavelet methods will be evaluated followed by the introduction, evaluation and comparison of the new algorithm, referred to as the “*eventogram*” throughout this paper. The *eventogram* provides and detects the dominant time-domain events within the biomedical signals using two moving averages and performs at a level that the traditional methods cannot achieve.

## 2. Materials and Methods

### 2.1. Data Used

**Figure 1:**
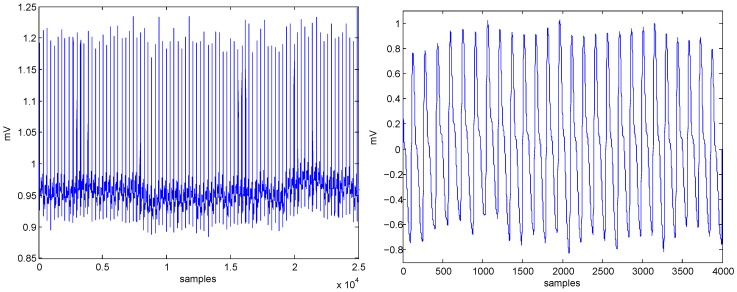
Data used in the analysis ECG signal (**left**) and PPG signal (**right**). Note, ECG stands for electrocardiogram while PPG stands for photoplethysmogram.

ECG and PPG signals were used for testing the algorithms, as shown in Figure 1. The ECG signal (sampling frequency of 360 Hz) was obtained from the MIT-BIH Arrhythmia Database [10], while the PPG signal (sampling frequency of 367 Hz) was obtained from the Heat Stress PPG Database [11].

### 2.2. Method I: Periodogram

Periodogram (also called spectral analysis) views the processed biomedical signal as a sum of cosine waves with varying amplitudes and frequencies. It is obtained by the fast Fourier transform and usually shows frequency as an *x*-axis, and magnitude of the spectrum as a *y*-axis. The main goal of the periodogram is to identify the important frequencies (or periods) in the processed biomedical signal. In other words, the periodogram graph shows the dominant frequency behavior within the signal. The periodogram, R(f), was calculated as follows:

(1) $$R\left(f\right)=\frac{1}{N}|\sum_{n=0}^{N-1}x_{n}e^{-i2\pi nf}{|}^{2},$$

where *x* is the biomedical signal with length *N*, *n* is the data sample, and *f* is the frequency.

### 2.3. Method II: Spectrogram

The main difference between the spectrogram and periodogram methods is that the spectrogram is a time-frequency visualization (takes into consideration time localization) while the periodogram relies only on the frequency domain. A spectrogram can be seen as multiple short periods of spectrum combined together. It is three-dimensional by nature, including the time, frequency, and magnitude of the spectrum. Typically, the spectrogram is displayed by using time as an *x*-axis and frequency as a *y*-axis. It also uses colors to show the magnitude of the spectrum. It is usually obtained by the short-time fast Fourier transform (FFT), which commonly includes a windowing function to extract the local time signal before conducting a short-time FFT. The main goal of the spectrogram is to identify the dominant frequencies with respect to time in the processed biomedical signal. The spectrogram, S(t,f), is calculated as follows:

(2) $$S\left(t,f\right)=|\int_{-\infty }^{\infty }x\left(\tau \right)W\left(\tau -t\right)e^{jf\tau }{d\tau |}^{2},$$

where *x* is the biomedical signal, W(τ) is the window function, and *f* is the frequency. The time index, *t*, is normally considered to be ‘slow’ time and is usually not expressed in as a high resolution as time *τ*.

### 2.4. Method III: Wavelets

A wavelet is the same as a spectrogram, but a wavelet transform can offer a multiresolution while the spectrogram can only offer a fixed resolution. Wavelets are closely related to filter banks. The wavelet transform (WT) of the biomedical signal x(t) is an integral transform defined by:

(3) $$D\left(a,b\right)=\int_{-\infty }^{\infty }x\left(t\right)\psi_{a,b}^{*}\left(t\right)\;dt,$$

where D(a,b) is known as the wavelet detail coefficient at the scale and location indices (a,b) of the biomedical signal x(t), and $\psi^{*}\left(t\right)$ denotes the complex conjugate of the wavelet function ψ(t). The transform yields a *time-scale* representation similar to the *time-frequency* representation of the short-time Fourier transform (STFT). In contrast to the STFT, the WT uses a set of analyzing functions that allow a variable time and frequency resolution for different frequency bands. The set of analyzing functions—the wavelet family $\psi_{a,b}\left(t\right)$—is deduced from a *mother wavelet* ψ(t) by:

(4) $$\psi_{a,b}\left(t\right)=\frac{1}{\sqrt{2}}\psi \left(\frac{t-b}{a}\right),$$

where *a* and *b* are the *dilation* (scale) and *translation* parameters, respectively. The scale parameter a of the WT is comparable to the frequency parameter of the STFT. The mother wavelet is a short oscillation with a zero mean. The orthonormal dyadic discrete wavelets are associated with scaling functions and their dilation equations. The scaling function is associated with the smoothing of the signal and has the same form as the wavelet, given by $\phi_{a,b}\left(t\right)=2^{-a/2}\phi \left(2^{-a}t-b\right)$. However, the convolution of the biomedical signal with the scaling function produces approximation coefficients as follows:

(5) $$A\left(a,b\right)=\int_{-\infty }^{\infty }x\left(t\right)\phi_{a,b}\left(t\right)\;dt,$$

where the biomedical signal x(t) can then be represented by a combined series expansion, using both the approximation coefficients *A* and the detail coefficients *D*.

### 2.5. Method IV: Eventogram

**Figure 2:**
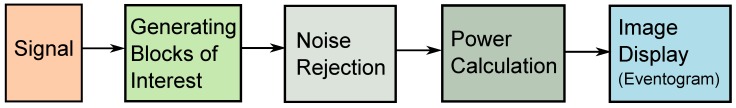
Flowchart for generating an *eventogram*. Generating an *eventogram* consists of four main stages: feature extraction (generating potential blocks using two moving averages), noisy events rejection, power calculation, and image display.

The *eventogram* is a novel visual-representation algorithm based on the fundamental concept of two moving averages. The idea of using two moving averages has already been tested over different biomedical signals for detecting *a* waves in APG (acceleration of PPG) signals [12,13,14], for detecting *c*, *d*, and *e* waves in APG [15], for detecting systolic waves in PPG signals [11], for detecting QRS complexes in ECG signals [16,17], for detecting T waves in ECG signals [18], and for detecting heart sounds [19]. An essential step of the two-moving-averages approach is the generation of the blocks of interest, and it is this step that is specifically applied in the *eventogram* methodology proposed in this paper. The blocks of interest are generated using two moving averages, which are now used to produce the *eventogram*. Noisy events rejection, power calculation, and image display are the new additional steps introduced to produce the *eventogram*. The structure of the *eventogram* is given in Figure 2.

**Generating Blocks** **of Interest:** Blocks of interest are generated using two event-related moving averages that demarcate the systolic and heartbeat areas. In this procedure, the first moving average (${\mathrm{MA}}_{1}$) is used to emphasize the first event and is given by

(6) $$\begin{array}{c} {\mathrm{MA}}_{1}\left[n\right]=\frac{1}{W_{1}}\left(y\left[n-\left(W_{1}-1\right)/2\right]+\cdots +y\left[n\right]+\cdots +y\left[n+\left(W_{1}-1\right)/2\right]\right), \end{array}$$

where $W_{1}$ represents the window size of the first-event duration. The resulting value is rounded to the nearest odd integer.

The second moving average (${\mathrm{MA}}_{2}$) is used to emphasize the second event area to be used as a threshold for the first moving average and is given by

(7) $$\begin{array}{c} {\mathrm{MA}}_{2}\left[n\right]=\frac{1}{W_{2}}\left(y\left[n-\left(W_{2}-1\right)/2\right]+\cdots +y\left[n\right]+\cdots +y\left[n+\left(W_{2}-1\right)/2\right]\right), \end{array}$$

where $W_{2}$ represents the window size of approximately one beat duration. Its value is rounded to the nearest odd integer.

In this stage, the blocks of interest are generated by comparing the ${\mathrm{MA}}_{1}$ signal with ${\mathrm{MA}}_{2}$. If any sample in ${\mathrm{MA}}_{1}\leq {\mathrm{MA}}_{2},$ it will be replaced by zero. If not, it will be replaced by a value of one. The generated time series of a binary string is called blocks of interest. However, some blocks of interest will contain noise segments that occur multiple times. The data within a series of ones are referred to as a *B*.

Since we obtained the blocks of interests, the next steps will start by rejecting the blocks that contain noise, calculating the power of each block, and then visualizing the power of the blocks in terms of $W_{1}$ and $W_{2}$, as follows:

**Noise** **Rejection:** Rejection is based on the average value of the standard deviations of all blocks. The standard deviation of each block (*m*) is calculated as follows:

(8) $$\sigma_{m}=\sqrt{\frac{1}{L}\sum_{n=1}^{L}{\left(B_{m}\left[n\right]-{\bar{B}}_{m}\right)}^{2}},$$

where *L* is the length of the data within each data block *B*.

After calculating the standard deviations of all blocks, a threshold (THR) is applied to reject blocks that contain noise as follows:

(9) $$\mathrm{THR}=\frac{1}{M}\sum_{m=1}^{M}\sigma_{m},$$

where *M* is the number of data blocks and THR is the average value of the standard deviations of all blocks. Usually, the noisy segments have small standard deviation compared to the ones that contain informative events (i.e., wave, spike, or peak). Thus, the block that satisfies σ>THR is accepted; otherwise, it is rejected.

**Power** **Calculation:** The power of each block, calculated at a specific window length $W_{1}$ and a specific window length $W_{2}$, is used to represent a value in the *eventogram*, as follows:

(10) $$P\left(W_{1},W_{2}\right)=\sum_{m=1}^{M}B_{m}^{2},$$

where *B* is the data segment within a specific block *m*. This step generates a power matrix: $P_{u\times v}$, where *u* is the different values of ($W_{1}$) and *v* is the number of values of ($W_{2}$).

**Image** **display:** The *P* matrix (obtained in the previous step) is displayed as an image with a full range of colors. Each element of *P* specifies the color for one pixel of the image representing the intensity of the power over the data block generated using $W_{1}$ and $W_{2}$. Here, the jet range is used from blue to red, and passes through the colors cyan, yellow, and orange. The blue color refers to the lowest value of power while the red color refers to the highest value of power. The resulting image is a *u*-by-*v* grid of pixels, where *u* is the number of columns $W_{1}$ and *v* is the number of rows $W_{2}$ in *P*. The visual representation of this step is called an *eventogram*—the image display step produces the *eventogram*. The main goal of the *eventogram* is to identify the dominant time-domain events and their associated signal morphologies within the processed biomedical signal using $W_{1}$ and $W_{2}$. The pixel associated with the highest value of power is considered to contain the main event. The *eventogram* is a time–time representation of a signal.

## 3. Results and Discussion

**Figure 3:**
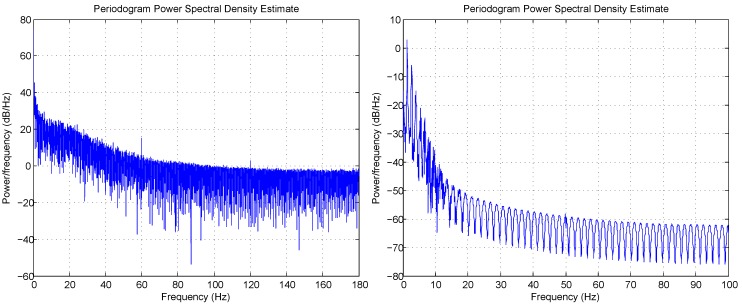
Periodogram for ECG signal (**left**) and PPG signal (**right**) shown in Figure 1.

**Figure 4:**
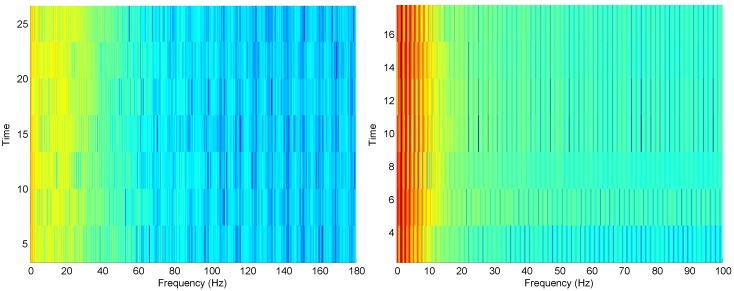
Spectrogram for ECG signal (**left**) and PPG signal (**right**) shown in Figure 1.

Figure 3 shows the periodogram for ECG and PPG; it provides frequency information about the processed signal. Both ECG and PPG examples have the direct current component and some low frequency noise. On the other hand, the spectrogram provides different frequency information about the signal, which is the frequency range that holds most of the energy of the processed signal. For example, most of the energy for the ECG signal lies in the range 0–30 Hz, while the PPG signal is in the range 0–10 Hz, as shown in Figure 4.

**Figure 5:**
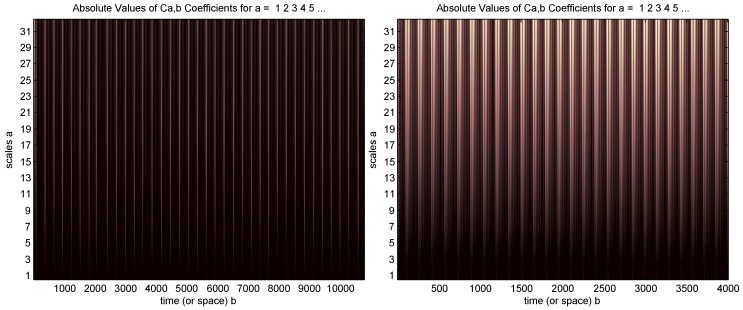
Wavelet for ECG signal (**left**) and PPG signal (**right**) shown in Figure 1.

A continuous wavelet transform is used with the “sym2” wavelet and a 1–32 scale based on the recommendation in [20], as shown in Figure 5. However, the wavelet visual presentation did not provide any information about the signal.

**Figure 6:**
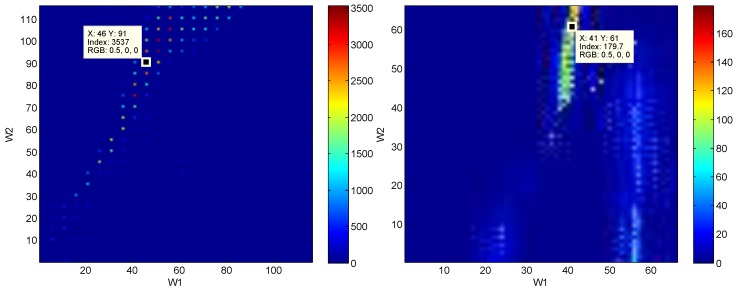
Eventogram for ECG signal (**left**) and PPG signal (**right**) shown in Figure 1. The **red** color indicates strong dominance of a main event while the **blue** color indicates non-existence of a main event. The *eventogram* is stored as a two-dimensional ($W_{1}$-by-$W_{2}$) array of integers in the range [1, length(colormap)]; colormap is a $W_{1}$-by-3 matrix of real numbers between 0.0 and 1.0. The *eventogram* returns the index value in terms of $W_{1}$ and $W_{2}$ for the most dominant event that is associated with the red color or **RGB** (0.5,0,0)—the **RGB** color model is an additive color model in which red, green, and blue light are added together in various ways to reproduce a broad array of colors.

Running the *eventogram* algorithm on the ECG and PPG signals (cf. Figure 6) provided useful information about the signals that was not provided by the traditional visualization methods. For example, in the ECG signal, $W_{1}$ and $W_{2}$ were varied and the maximum power value was found at window size $W_{1}=46$ samples and $W_{2}=91$ samples. This means there is a dominant event of length 46 samples occurring within a periodic event of length 91 samples. For PPG signals, the window sizes ($W_{1}$ and $W_{2}$) were also varied, and the maximum power value was found at $W_{1}=41$ samples and $W_{2}=61$ samples. However, the traditional visualization methods did not provide any insights into the events within the ECG and PPG signals.

The *eventogram* describes the biomedical signal in terms of two time-domain window sizes $W_{1}$ and $W_{2}$. The *eventogram* is a time–time representation which is different from the time-frequency representation produced by the spectrogram and wavelet methods. The time–time representation of a biomedical signal in terms of two windows will help clinicians understand the dominant durations and visualize their morphologies. Thus, the *eventogram* is more informative than the classical visualization methods. Note, the Recurrence Plot [21] was not included in this study, as it represents phase space trajectory of the processed signal. The phase analysis is not meaningful for physicians or biomedical engineers when analyzing and diagnosing biomedical signals—correlating phase changes with a disease is not easy nor precise.

**Figure 7:**
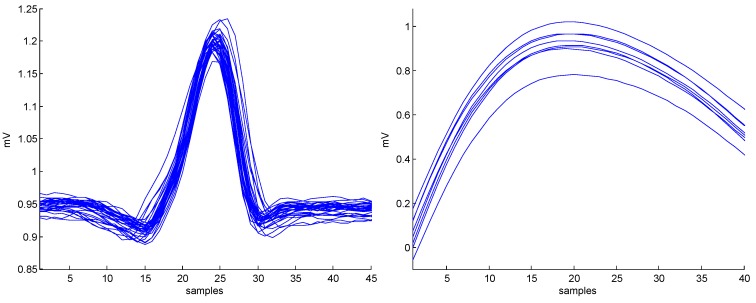
The main events (superimposed) detected by the *eventogram*s shown in Figure 6 based on the highest value of power scored with $W_{1}$ and $W_{2}$ for the ECG (**left**) and the PPG (**right**) signals shown in Figure 1. Note, ECG stands for electrocardiogram while PPG stands for photoplethysmogram.

The application of the *eventogram* methodology on PPG and ECG signals is demonstrated in Figure 6. Dominant events are marked by red pixels where the combination of $W_{1}$ and $W_{2}$ scored the highest power. Once $W_{1}$ and $W_{2}$ are identified for the most dominant event, the next step is to visualize its corresponding waveform in terms of duration and morphology, as shown in Figure 7. In the case of ECG signal, the *eventogram* picked the QRS complexes to be the most dominant event, while the *eventogram* detected the systolic wave as the most dominant event in the PPG signal. Note that examination of the second highest power, the third highest, and so on, in the *eventogram* is also possible to check their corresponding dominant events.

**Figure 8:**
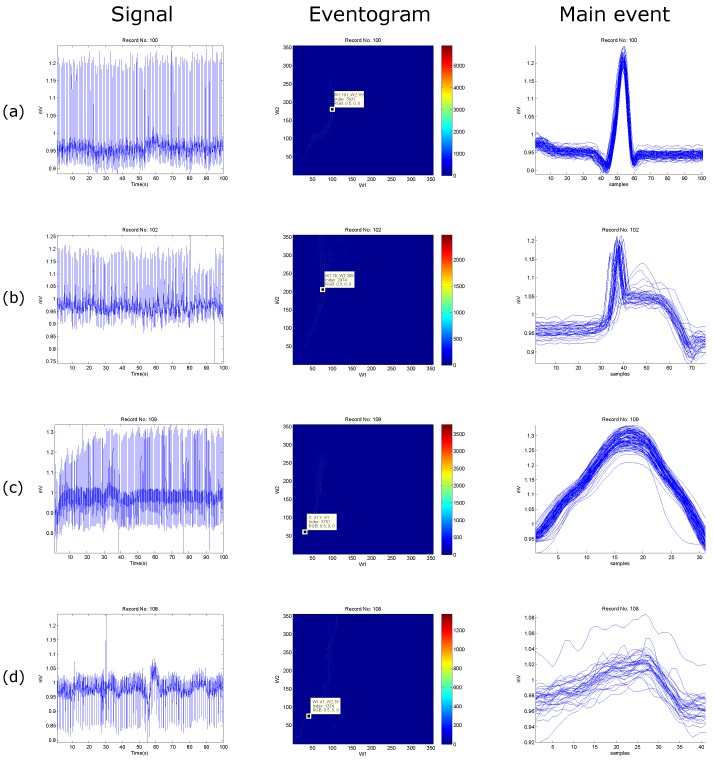
Examples of *eventogram* output using ECG signals. (**a**) normal beats; (**b**) paced beats; (**c**) left bundle branch block beats; and (**d**) noisy ECG signal. The **red** color indicates strong dominance of a main event while the **blue** color indicates non-existence of a main event. The *eventogram* is stored as a two-dimensional ($W_{1}$-by-$W_{2}$) array of integers in the range [1, length(colormap)]; colormap is a $W_{1}$-by-3 matrix of real numbers between 0.0 and 1.0. The *eventogram* returns the index value in terms of $W_{1}$ and $W_{2}$ for the most dominant event that is associated with the red color or RGB (0.5,0,0).

**Figure 9:**
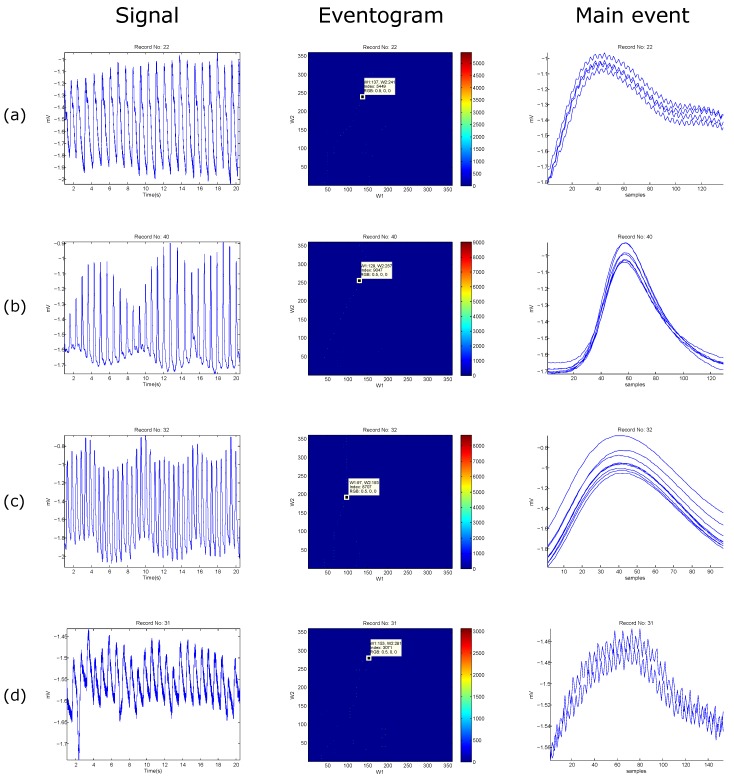
Examples of *eventogram* output using PPG signals. (**a**) PPG waveforms with Salient dicrotic notch; (**b**) PPG waveforms with a nonsalient dicrotic notch; (**c**) PPG signal measured after simulated heat stress; and (**d**) noisy PPG signal. The **red** color indicates strong dominance of a main event while the **blue** color indicates non-existence of a main event. The *eventogram* is stored as a two-dimensional ($W_{1}$-by-$W_{2}$) array of integers in the range [1, length(colormap)]; colormap is a $W_{1}$-by-3 matrix of real numbers between 0.0 and 1.0. The *eventogram* returns the index value in terms of $W_{1}$ and $W_{2}$ for the most dominant event that is associated with the **red** color or RGB (0.5,0,0).

Another advantage of the *eventogram* is that the search area can be either customizable or automated. For example, the search ranges for the ECG signal were 1–115 samples for $W_{1}$ and $W_{2}$, while 1–65 samples for the PPG signal. In Figure 8 and Figure 9, the search areas are automatically adjusted to equal the sampling frequency, and the step sizes are automatically adjusted to be the log of the sampling frequency—the value will then be rounded toward negative infinity.

The window lengths for the ECG signals (cf. Figure 8) are 360 samples with a step size of five samples for ECG data length of 100 s, while the window lengths for the PPG signals (cf. Figure 9) are 367 samples with a step size of five samples for PPG data length of 20 s.

The *eventogram* analysis of biomedical signals with regular heart rhythm is simple and efficient as the heart beats are repeated with an equally spaced pattern. This regularity helps the time-domain threshold methodologies to detect main events successfully. The regular heart rhythm is called the normal sinus rhythm [22], which means that the rhythm is constant and the occurrence of the next beat is predictable. The *eventogram* easily detects the QRS complexes and systolic peaks correctly in a regular heart rhythm in ECG and PPG signals, respectively, as shown in Figure 8a and Figure 9a. Moreover, the *eventogram* is able to detect main events in biomedical signals with irregular rhythm, as shown in Figure 8b,c and Figure 9c. The results show that the *eventogram* is suitable for any quasi-periodic biomedical signals, as it is robust against the irregular rhythm, non-stationary effects (cf. Figure 8b–d and Figure 9b–d), and low signal-to-noise ratio (cf. Figure 8d and Figure 9d).

Classical visualization methods do not provide precise information in terms of event morphology and duration within the processed signal. These methods rely only on qualitative examination, compared to the proposed *eventogram* method that relies on both qualitative and quantitative analysis. The *eventogram* uses different window size choices, where dominant events can be quantitatively and qualitatively visualized, therefore providing more precise insight into signal characteristics. To validate the concept, application, and performance of the *eventogram*, rigorous testing over multiple data sets for detecting different events were conducted, as follows:

- **For QRS detection in ECG signals:** The *eventogram*-based QRS detector has two additional steps to the *eventogram*: one at the beginning before applying the *eventogram* (preprocessing step) and one at the end after applying the *eventogram* (thresholding step). The *eventogram* is considered to be the feature extraction step. Interestingly, the *eventogram*-based QRS detector, with $W_{1}=97$ ms and $W_{2}=611$ ms, obtained a sensitivity (SE) of 99.29% and a positive predictivity (+P) of 98.11% over the first lead of 10 databases with a total of 1,179,812 beats. When applied to the well-known MIT-BIH Arrhythmia Database, an SE of 99.78% and a +P of 99.87% were attained [17].
- **For T wave detection in ECG signals:** The *eventogram*-based T wave detector has three additional steps to the *eventogram*: two steps at the beginning before applying the *eventogram* (filtering and QRS removal) and one at the end after applying the *eventogram* (thresholding based on RR intervals). The *eventogram* is considered to be the feature extraction step. Over the MIT-BIH Arrhythmia Database, the *eventogram*-based T wave detector, with $W_{1}=70$ ms and $W_{2}=140$ ms, achieved a SE of 99.86% and a +P of 99.65%, which are promising results for handling the non-stationary effects, low SNR, normal sinus rhythm (NSR), left bundle branch block, right bundle branch block, premature ventricular contraction, and premature atrial contraction in ECG signals [18].
- **For systolic wave detection in PPG signals:** The *eventogram*-based systolic wave detector has two additional steps to the *eventogram*: one step at the beginning before applying the *eventogram* (filtering) and one at the end after applying the *eventogram* (thresholding). The *eventogram* is considered to be the feature extraction step. The *eventogram*-based systolic wave detection algorithm, with $W_{1}=111$ ms and $W_{2}=667$ ms, was evaluated using 40 records after three heat stress simulations, containing 5071 heartbeats, with an overall SE of 99.89% and the +P was 99.84% [11].
- **For a and b wave detection in PPG signals:** The *eventogram*-based *a* and *b* waves detector has two additional steps to the *eventogram*: one step at the beginning before applying the *eventogram* (filtering) and one at the end after applying the *eventogram* (thresholding). The *eventogram* is considered to be the feature extraction step. The *eventogram*-based *a* wave detection algorithm, with $W_{1}=175$ ms and $W_{2}=1000$ ms, demonstrated overall SE of 99.78%, +P of 100% over signals that suffer from (1) non-stationary effects; (2) irregular heartbeats; and (3) low amplitude waves. In addition, the *b* detection algorithm (based on the detection of *a* waves) achieved an overall SE of 99.78% and a +P of 99.95% [14].
- **For c, d, and e wave detection in PPG signals:** The *eventogram*-based *c*, *d*, and *e* waves detector has three additional steps to the *eventogram*: two steps at the beginning before applying the *eventogram* (filtering and removal of ab segment) and one at the end after applying the *eventogram* (thresholding). The *eventogram* is considered to be the feature extraction step. The performance of the *eventogram*-based *c*, *d*, and *e* waves detector, with $W_{1}=5$ ms and $W_{2}=15$ ms, was tested on 27 PPG records collected during rest and after two hours of exercise, resulting in 97.39% SE and 99.82% +P [15].
- **For S1 and S2 detection in heart sounds:** The *eventogram*-based heart sounds detector has two additional steps to the *eventogram*: one step at the beginning before applying the *eventogram* (filtering) and one at the end after applying the *eventogram* (thresholding). The *eventogram* is considered to be the feature extraction step. The SE and +P of the *eventogram*-based S1 and S2 detector, with $W_{1}=130$ ms and $W_{2}=270$ ms, were 70% and 68%, respectively, in heart sounds collected from children with pulmonary artery hypertension [19].

Finally, *eventogram*-based detectors succeeded in detecting QRS and T waves in ECG signals, systolic and a,b,c,d,e waves in PPG, and S1 and S2 in heart sounds. Results show that the *eventogram*-based detectors are promising in terms of computational complexity and efficiency. This paper is considered to be a proof-of-concept work. The proposed visualization method is simple and efficient when applied to ECG and PPG signals; however, it may fail when applied to other signals. As it is a new concept, there is a need to publish the current results and let the scientific community evaluate its performance on their studies.

Presently, the *eventogram* is tested over one-dimensional biomedical signals collected and analyzed independently, covering temporal and spatial visualization. However, for multiple signals collected simultaneously, there will be a need to test the vectorial and interactive visualizations capability of the *eventogram*. Note that vectorial visualizations present signals with respect to direction and magnitude, while interactive visualization facilitates efficient interrogation of biomedical signals at different levels of detail. As the core implementation of the *eventogram* relies mainly on two simple moving averages (fast algorithms), it is expected that there will be no issue in terms of computational efficiency with vectorial and interactive visualizations. Because of the *eventogram*’s intuitive visualization and computational efficiency, it can be implemented on mobile devices.

The significance of the *eventogram* comes from incorporating two types of visualization: temporal and spatial. Note that temporal visualizations present signals with respect to time, while spatial visualizations present data in 2D or 3D space. Moreover, the design and implementation of the *eventogram* set a framework that can be modified in a standard way, as suggested in [23]. In other words, we can consider the *eventogram* as LEGO building blocks. The *eventogram* has three main building blocks: generating blocks of interest, noise rejection, and power calculation. Each one of these steps can be modified independently based on the detection problem, which makes the *eventogram* more flexible, universal, and applicable to achieve high accuracy.

The *eventogram* offers the advantages of being used as is or can be customized by researchers to build a new and better *eventogram* following the LEGO analogy (e.g., change the filter type, filter order, moving average type based on their applications). The use of moving averages provides efficient temporal filtering to the processed signal where it is statistically stationary at least over the duration of the moving window. Exploring these findings across different types of signals, such as the optical signal, the communication signal, the geophysical and astrophysical signal, the earth resources signal, the acoustic and vibration signal, the radar signal, and the sonar signal, will improve generalization across the entire signal analysis discipline.

## 4. Conclusions

Event detection in biomedical signals is an important step before analyzing the corresponding waveform in more detail. A newly introduced visualization algorithm, referred to as the *eventogram*, depends on two event-related moving averages. The *eventogram* provides us with important information about the signal, such as the duration of the most dominant event and its morphology. It also provides new information and insight into the processed signal when compared to the traditional signal visualization techniques. Dominant events identified by the *eventogram* will provide a clearer set of information for clinicians to improve their decision making. Moreover, the performance of the *eventogram*-based detector is promising, as it has been tested on different databases that contain unusual noise and different waveform morphologies. One of the unique and powerful characteristics of the *eventogram* is its ability to be applied to different types of biomedical and quasi-periodic signals.
